# TR4 Nuclear Receptor Different Roles in Prostate Cancer Progression

**DOI:** 10.3389/fendo.2015.00078

**Published:** 2015-05-27

**Authors:** Shin-Jen Lin, Dong-Rong Yang, Gonghui Li, Chawnshang Chang

**Affiliations:** ^1^George Whipple Lab for Cancer Research, Departments of Pathology, Urology, Radiation Oncology and the Wilmot Cancer Center, University of Rochester Medical Center, Rochester, NY, USA; ^2^Department of Urology, The Second Affiliated Hospital of Soochow University, Soochow, China; ^3^Department of Urology, Sir-Run Run Shaw Hospital, Zhejiang University, Hangzhou, China; ^4^Sex Hormone Research Center, China Medical University Hospital, Taichung, Taiwan

**Keywords:** TR4, prostate cancer, ATM, PPAR, TZD

## Abstract

Nuclear receptors are important to maintain the tissue homeostasis. Each receptor is tightly controlled and under a very complicated balance. In this review, we summarize the current findings regarding the nuclear receptor TR4 and its role in prostate cancer (PCa) progression. In general, TR4 can inhibit the PCa carcinogenesis. However, when PPARγ is knocked out, activation of TR4 can have an opposite effect to promote the PCa carcinogenesis. Clinical data also indicates that higher TR4 expression is found in PCa tissues with high Gleason scores compared to those tissues with low Gleason scores. *In vitro* and *in vivo* studies show that TR4 can promote PCa progression. Mechanism dissection indicates that TR4 inhibits PCa carcinogenesis through regulating the tumor suppressor ATM to reduce DNA damages. On the other hand, in the absence of PPARγ, TR4 tends to increase the stem cell population and epithelial–mesenchymal transition (EMT) *via* regulating CCL2, Oct4, EZH2, and miRNA-373-3p expression that results in increased PCa carcinogenesis. In opposition to PCa initiation, TR4 can increase PCa metastasis *via* modulating the CCL2 signals. Finally, targeting TR4 enhances the chemotherapy and radiation therapy sensitivity in PCa. Together, these data suggest TR4 is a key player to control PCa progression, and targeting TR4 with small molecules may provide us a new and better therapy to suppress PCa progression.

## Introduction

The testicular nuclear receptor 4 (TR4) was first cloned in 1994, and was identified as one of critical nuclear receptors to maintain the physiological homeostasis (1). As a transcription factor, TR4 binds to the direct repeat sequence (AGGTCA with spacings of 0–6) to regulate its target genes transcription (2, 3). The upstream modulators including natural polyunsaturated fatty acids (PUFA) or synthetic thiazolidinediones (TZD) can activate TR4 function (4, 5). Phosphorylation (6, 7) or acetylation (8) of TR4, on the other hand, will inhibit its activity. As a result, many physiological functions are maintained and regulated including metabolism, fertility, bone formation, and neural development in the whole body (7, 9–16). In this review, we will summarize the current findings of TR4 roles in prostate cancer (PCa) progression.

## TR4 Role in PCa Initiation

Both *in vivo* and *in vitro* studies indicated that TR4 acts as a caretaker tumor suppressor that suppresses PCa initiation through promoting DNA repair and maintaining genome integrity (17). *In vivo* mouse data showed prostatic intraepithelial neoplasia (PIN) was found in prostates of old TR4 knockout (TR4^-/-^) mice, while their wild type TR4 (TR4^+/+^) littermates showed normal prostate histology. Furthermore, both PTEN^+/-^ and TRAMP PCa mouse models showed one allele deletion of TR4 could accelerate the PCa initiation. In the PTEN^+/-^/TR4^+/-^ model, the mice not only form PIN as expected in PTEN^+/-^/TR4^+/+^ mice, but also form PCa tumors at 15 months old that does not happen in the PTEN^+/-^/TR4^+/+^ mice (17). In the TRAMP mouse model, TR4^+/-^/TRAMP mice can form tumors or severe PIN as early as 24 weeks while the TR4^+/+^/TRAMP mice only form minor PIN. In summary, three mouse models all confirm that loss of TR4 will accelerate the PCa initiation.

*In vitro* data also support the *in vivo* findings showing that two normal prostate epithelial cell lines can be transformed more easily when TR4 is knocked-down by TR4-shRNA (17). Mechanistically, Lin et al. found the DNA repair gene *ATM* can be regulated by TR4 at the transcription level. The two PCa mouse models and two cell lines showed ATM was reduced dramatically, with DNA damage increased, when TR4 is reduced by one allele deletion or knocked-down by TR4-shRNA (17). Finally, the clinical data also revealed that ATM expression is highly correlated with TR4 expression in PCa tissues (17). Together, these findings suggest TR4 can prevent PCa initiation through maintaining DNA integrity.

## PPARγ and TR4 Interplay in PCa Initiation

Lin et al. analyzed Peroxisome Proliferator-Activated receptor gamma *(PPAR*γ) gene deletion in 69 human PCa samples with fluorescent *in situ* hybridization (FISH) assays, and results showed 9% of PCa samples have one allele *PPAR*γ deletion. In contrast, there was no *PPAR*γ deletion in their normal prostate compartment (Fisher’s test, *p* = 0.0279), suggested the deletion of *PPAR*γ may have some linkage to the PCa initiation (18).

Early reports found that TZDs, the agonists of *PPAR*γ might be also able to activate TR4, suggesting a potential cross-talk might exist between these two nuclear receptors (5). It would be interesting to see if TR4 might exert different effects in PCa cells with or without *PPAR*γ deletion. Lin et al. assayed the cell proliferation in *PPAR*γ naïve mPrE (mPrE^+/+^) versus *PPAR*γ knockout mPrE prostate cells (mPrE^-/-^). The results showed knocking down TR4 suppressed cell proliferation in mPrE^-/-^ cells under carcinogen treatment, which are opposite the results found in the mPrE^+/+^ cells (17). Next, they found overexpression of TR4 promoted cell proliferation in mPrE^-/-^ cells under carcinogen treatment. Together, the *in vitro* results suggest TR4 has different functions in the presence or absence of *PPAR*γ that can promote prostate epithelial cell growth.

Lin et al. then used different assays to test whether the status of *PPAR*γ deletion may also influence TR4 effect on PCa initiation. Using cell transformation with colony formation assays, they found knocking-down TR4 suppressed, while overexpression of TR4 promoted PCa carcinogenesis in mPrE^-/-^ cells, which is also opposite from the mPrE^+/+^ cells data (17). Together, results support the above findings that different TR4 effects on prostate epithelium cell proliferation and PCa initiation are dependent on the deletion status of *PPAR*γ (17, 18).

Another approach that may be similar to deleting *PPAR*γ is to apply an antagonist to suppress PPARγ activity. Lin et al. treated mPrE^+/+^ cells with *PPAR*γ inhibitor GW9662 and assayed the cell growth by MTT assay. The results showed knocking down TR4 in mPrE^+/+^ cells treated with GW9662 suppressed cell proliferation, which is consistent with the mPrE^-/-^ cell data above. Together, the results confirm the above conclusion that TR4 may exert different effects on prostate cell proliferation depending on the PPARγ status that either was deleted or suppressed by its antagonists (18).

To prove the above *in vitro* cell lines data *in vivo*, Lin et al. applied a xenograft mouse model to test whether TR4 could also promote mPrE^-/-^ tumor growth *in vivo*. TR4 was either overexpressed or knocked-down in mPrE^-/-^ cells, and a total of 10^6^ cells were then subcutaneously injected into the dorsal flank of the mice. The PCa mass in mice injected with overexpressed TR4 cells were much larger than those in the control group at 7 weeks. In contrast, at 14 weeks, much smaller PCa masses were seen in the mice injected with TR4 knocked-down cells. These *in vivo* results therefore confirmed the *in vitro* data showing overexpressing TR4 could lead to PCa tumor growth in mPrE^-/-^ cells xenografts (18).

The morphology showed that overexpressing TR4 increased prostate de-differentiation in mPrE^-/-^ cells. In contrast, knocking-down TR4 failed to show such prostate de-differentiation in mPrE^-/-^ cells, suggesting TR4 might regulate PCa growth in the absence of PPARγ by mediating PCa to a more poorly differentiated type of cancer. Molecular mechanism dissection found overexpressing TR4 in mPrE^-/-^ cells enhanced the expression of CD44 and Scal1, but eliminated the expression of E-cadherin, indicating an epithelial–mesenchymal transition (EMT) in the stem cell-like population among PCa cells (18).

## TZD Effect on TR4 in PCa Progression

Thiazolidinediones (TZD) are potent anti-diabetic drugs that function through targeting PPARγ (19). An earlier report showed that TZD could also activate TR4 (5). Lin et al. found 9% of PCa patients have the *TR4* gene deletion. It is important to test whether TZD treatment has different effects between tumors/cells with wild type TR4 versus TR4 deletion. They first applied TR4–shRNA to knock-down TR4 in PCa CWR22RV1 cells to mimic the TR4 deletion and then treated with TZD. Interestingly, they found TZD treatment could promote CWR22RV1 cell growth significantly compared to vehicle control. In contrast, TZD showed no effect on CWR22RV1 cell growth compared to vehicle control when cells were transfected with scramble shRNA (20).

Lin et al. then applied the second growth assay, an anchorage independent assay also known as colony formation assay, to test TZD differential effects. As expected, TZD treatment could also dramatically increase colony formation in CWR22RV1 cells where TR4 is knocked down compared to vehicle control. In contrast, TZD treatment decreased colony formation on CWR22RV1 cells transfected with scramble shRNA compared to vehicle control. They also confirmed these different *in vitro* phenotypes using another PCa cell line, C4-2, and obtained similar results (20). Together, the results suggest that TZD treatment may have different effects on PCa progression that depends on the TR4 expression status.

Lin et al. were also interested to see if TZD treatment may have different effects on PCa metastasis, and therefore applied the Boyden chamber migration/invasion assays to examine the CWR22RV1 metastatic ability. TZD treatment increased CWR22RV1_shTR4 migration significantly compared to vehicle control. In contrast, TZD treatment decreased CWR22RV1_scr migration compared to vehicle control. For the invasion assays, they pre-coated membranes between upper and bottom wells with matrigel to mimic *in vivo* invasion, as the cancer cells need to invade into the extracellular matrix. TZD treatment increased CWR22RV1_shTR4 invasion significantly compared to vehicle control. In contrast, TZD treatment had no effect on CWR22RV1_scr invasion compared to vehicle control. They also confirmed these different *in vitro* phenotypes using C4-2 cells, and obtained similar results (20). Together, the results suggest that TZD treatment may also have different effects on PCa metastasis that depends on the TR4 expression status.

To confirm the *in vitro* finding *in vivo*, Lin et al. xenografted TZD treated CWR22RV1_scr and CWR22RV1_shTR4 cells into nude mice prostate. They sacrificed the mice at 4 weeks after inoculation and found the CWR22RV1_shTR4 group grew larger tumors than the CWR22RV1_scr group. Furthermore, they found CWR22RV1_shTR4 group had more metastasis than CWR22RV1_scr group (20). Together, the results conclude that TZD treatment has adverse effects on PCa progression *in vivo* when TR4 is low, and suggests treating those diabetic PCa patients who lost one allele of TR4 with TZD may lead to PCa progression.

## TZD has Different Effects on HRAS Expression

Lin et al. then wanted to know what is downstream of TZD to induce PCa progression. They screened 35 metastasis-related genes and found HRAS mRNA increased in both PCa cell lines, CWR22RV1_shTR4 and C4-2_shTR4, treated with TZD, but decreased in the scramble controls treated with TZD. Furthermore, they confirmed TZD could increase *HRAS* promoter activity by luciferase-reporter assay when TR4 is knocked down (20). These findings fit the phenotypes showing TZD treatment has differential effects depending on the TR4 status.

## HRAS Specific Inhibitor Interrupts TZD Induced Cell Migration and Invasion

After finding the potential candidate HRAS, Lin et al. tried to inhibit HRAS by treating with the specific inhibitor farnesyl thiosalicylic acid (FTS). They treated the four sub-clones (scramble shRNA_DMSO, scramble shRNA_TZD, TR4 shRNA_DMSO, and TR4 shRNA_TZD) of CWR22RV1 and C4-2 cells with 50 μM FTS for 2 weeks. After treatment, they assayed the cell migration and invasion as described above. The results showed FTS could successfully interrupt TZD induced migration and invasion in both cell lines (20).

## Conclusion of TR4, PPARγ, and TZD

The interaction between TR4 and PPARγ in PCa is more complicated than we expected. In the absence of *PPAR*γ, TR4 becomes an oncogene instead of a tumor suppressor gene. Reciprocally, in the absence of TR4, *PPAR*γ becomes an oncogene instead of a tumor suppressor gene through TZD signaling. Recently, the two major TZD anti-diabetic drugs, Avandia (rosiglitazone) and Actos (pioglitazone), have been withdrawn or received warnings against use by the FDA, respectively. The major warning for Actos use is that long-term treatment may increase bladder cancer risk. Yang et al. found 38% of bladder cancer patients have a *PPAR*γ gene amplification, which indicates the unbalance between TR4 and PPARγ plays an important role in both PCa and bladder cancer (21).

After screening 35 metastasis-related genes expression in PCa cell lines, Lin et al. found TZD treatment can increase HRAS mRNA level only when TR4 is reduced. In contrast, TZD did not increase, but decreased HRAS mRNA level when TR4 is normal. Some reports have demonstrated *HRAS* is not only the oncogene for cancer initiation, but also can increase PCa metastasis (22, 23).

Together, the results of this study conclude that TR4 is a key regulator to prevent TZD from being oncogenic. Patients with TR4 deletion should be warned about taking TZD, or consider combining TZD treatment with FTS to eliminate the side effects.

## TR4 Role in PCa Metastasis

Opposite to the PCa initiation, Ding et al. found TR4 could promote PCa metastasis (24). From the clinical PCa tissue staining results, Ding et al. showed higher expression of TR4 with high Gleason scores compared to the tissues with the low Gleason scores. *In vitro* assays showed that TR4 promoted PCa cells migration/invasion. Mechanism dissection using QPCR and ELISA found that the CCL2 signaling was increased, which may contribute to the mediation of TR4-promoted PCa cells migration/invasion. Chromatin immunoprecipitation (ChIP) and luciferase assays further confirmed TR4 modulation of CCL2 at the transcriptional level, and addition of the CCL2 receptor antagonist interrupted the TR4-enhanced PCa cells migration/invasion. Finally, the orthotopic xenografted mice model confirmed that TR4 did enhance PCa metastasis, and the metastasis was alleviated when the mice were treated with the CCL2 receptor antagonist. Together, the clinical data as well as the *in vitro* and *in vivo* results revealed a positive TR4 role in PCa metastasis, which may function through CCL2 signaling, and targeting the TR4-CCL2 axis may become a new therapeutic approach to battle PCa metastasis (24).

Another report revealed that TR4 might enhance PCa cells invasion *via* inhibition of the microRNA-373-3p (miR-373-3p) expression, and TR4-enhanced PCa cells invasion can be interrupted by adding back the miR-373-3p (25). Mechanistically, they found that miR-373-3p functions through inhibition of the TGFβR2→p-Smad3 signals to inhibit the PCa cells invasion. The *in vivo* mouse model using orthotopic xenografts also confirmed that TR4 enhanced PCa metastasis is through the inhibition of miR-373-3p. The data suggests that TR4 increases the PCa metastasis through the signaling of miR-373-3p, and using the TR4 antagonist, TR4-siRNA, or miR-373-3p may become a new potential therapeutic approach to better suppress PCa metastasis (25).

Moreover, Zhu et al. demonstrated that TR4 plays a positive role in PCa stem/progenitor (S/P) cell invasion (26), and targeting TR4 with shRNA significantly suppressed PCa S/P cell invasion both *in vitro* and *in vivo*. Mechanism dissection using ChIP and luciferase assays found that TR4 transcriptionally regulates the oncogene *EZH2*. EZH2 may then regulate the expression of its downstream key metastasis-related genes including *NOTCH1, TGF*β*1, SLUG*, and *MMP9*. Adding back EZH2 in the TR4–shRNA PCa cell lines can partially interrupt the TR4–shRNA suppression effect on PCa cells invasion. Together, these results suggest that EZH2 is another TR4 target that plays a critical role in the PCa S/P cell invasion (26).

## TR4 Role in PCa Chemotherapy and Radiation Therapy

Zhu et al. demonstrated that TR4 plays a protective role against ionizing radiation (IR) in PCa cells, and targeting TR4 with shRNA enhances the IR sensitivity in PCa cells. Mechanism dissection found that targeting TR4 by shRNA enhanced miR-212-3p expression *via* de-methylation of the promoter region. Enhanced miR-212-3p then suppressed the expression of DNA damage/repair related gene *BRCA1via* binding to the BRCA1 mRNA 3′ un-translated region. Furthermore, the TR4–shRNA effect on enhancing PCa radiation sensitivity can be interrupted by adding miR-212-3p inhibitor. Together, these results conclude that targeting TR4 can increase the IR sensitivity in PCa (26).

Prostate cancer S/P cells are known to have higher chemoresistance than non-S/P cells, but the underlying molecular mechanism remains unclear (27). Yang found the expression of TR4 is significantly higher in PCa S/P cells. Knocking down TR4 in the PCa S/P cells with shRNA led to increased chemosensitivity to docetaxel and etoposide. Mechanism dissection showed that suppression of TR4 down-regulated the Oct4 expression, which, in turn, down-regulated the IL-1 receptor antagonist (IL1Ra) expression. To confirm this, Yang et al. performed the interruption assay by over-expression of Oct4 or adding back the IL1Ra recombinant, and found TR4–shRNA could no longer increase the chemosensitivity (28). Together, these studies suggest that targeting TR4 can alleviate the chemoresistance of PCa, therefore improve chemotherapy efficacy.

## Conclusion

**Figure 1:**
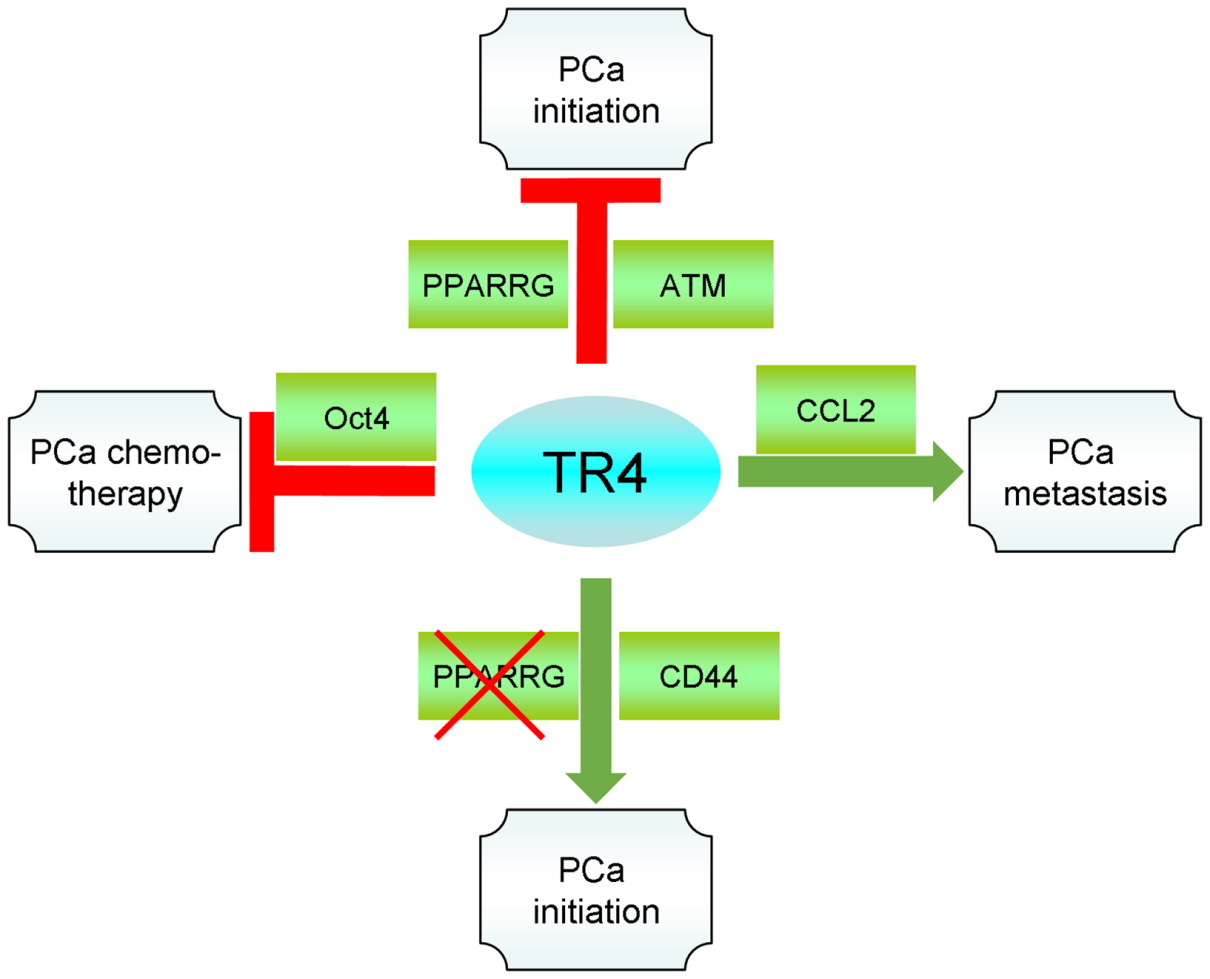
**Summary of TR4 role in PCa**.

TR4, as a transcription factor, plays multiple functions in PCa through regulating a variety of downstream targets. In the PCa initiation, TR4 can prevent DNA damage by regulating the DNA repair genes *ATM* and *BRCA1*. When PPARγ is absent, TR4 tends to regulate the EMT with increased S/P population and therefore increases the PCa initiation. On the other hand, when TR4 is knocked-down by shRNA, the TR4 and PPARγ shared activator/ligand, TZD, exclusively enhances PPARγ activity, which, in turn, increases PCa progression. When the PCa is developed, TR4 now becomes an activator to promote PCa cells invasion/metastasis. Increase of CCL2, decrease of miR-373-3p, and increase of EZH2 by TR4 all contribute to the increased PCa invasion/metastasis. Finally, TR4 also contributes to the radiation therapy resistance and chemotherapy resistance. TR4 inhibits miR-212-3p that enhances BRCA1 expression in the PCa cell lines that contributes to the radiation resistance. TR4 can also increase Oct4, and therefore increase the S/P population that contributes to the chemoresistance. Together, activation of TR4 may be beneficial to prevent the PCa initiation in patients in which the expression of PPARγ is normal, while inhibiting TR4 *via* siRNA may be beneficial to prevent the PCa metastasis (Figure 1).

## Conflict of Interest Statement

The authors declare that the research was conducted in the absence of any commercial or financial relationships that could be construed as a potential conflict of interest.
